# The Roles of BDNF, pCREB and Wnt3a in the Latent Period Preceding Activation of Progenitor Cell Mitosis in The Adult Dentate Gyrus by Fluoxetine

**DOI:** 10.1371/journal.pone.0013652

**Published:** 2010-10-27

**Authors:** Scarlett B. Pinnock, Alastair M. Blake, Nicola J. Platt, Joe Herbert

**Affiliations:** Department of Physiology, Development and Neuroscience and Cambridge Centre for Brain Repair, Department of Clinical Neurosciences, University of Cambridge, Cambridge, United Kingdom; Universidade Federal do Rio de Janeiro, Brazil

## Abstract

The formation of new neurons continues into adult life in the dentate gyrus of the rat hippocampus, as in many other species. Neurogenesis itself turns out to be highly labile, and is regulated by a number of factors. One of these is the serotoninergic system: treatment with drugs (such as the SSRI fluoxetine) markedly stimulates mitosis in the progenitor cells of the dentate gyrus. But this process has one remarkable feature: it takes at least 14 days of continuous treatment to be effective. This is despite the fact that the pharmacological action of fluoxetine occurs within an hour or so of first administration. This paper explores the role of BDNF in this process, using the effect of a Trk antagonist (K252a) on the labelling of progenitor cells with the mitosis marker Ki67 and the associated expression of pCREB and Wnt3a. These experiments show that (i) Fluoxetine increased Ki67 counts, as well as pCREB and Wnt3a expression in the dentate gyrus. The action of fluoxetine on the progenitor cells and on pCREB (but not Wnt3a) depends upon Trk receptor activation, since it was prevented by icv infusion of K252a. (ii) These receptors are required for both the first 7 days of fluoxetine action, during which no apparent change in progenitor mitosis occurs, as well as the second 7 days. Increased pCREB was always associated with progenitor cell mitosis, but Wnt3a expression may be necessary but not sufficient for increased progenitor cell proliferation. These results shed new light on the action of fluoxetine on neurogenesis in the adult dentate gyrus, and have both clinical and experimental interest.

## Introduction

The realisation that neurogenesis continues in the dentate gyrus into adult life has aroused considerable interest for several reasons. The functional significance of a generative and restorative process, more typical of the developing brain, in this region of the adult remains enigmatic. It has also raised hopes that a similar process might be inducible in other areas following damage or malfunction. A number of attempts, some more convincing than others, have been made to show that inhibiting neurogenesis - a procedure which has technical problems related to its specificity - reduces mnemonic abilities [1], [2], [3], [4] though precisely why this should require newly-formed neurons is still debated [5], [6]. There is the suggestion that the onset of depression in humans, or its response to treatment with antidepressants, may be related in some way to altered neurogenesis in the dentate gyrus[7], [8], [9]. Direct evidence for this hypothesis is still scanty and conflicting [10], [11]. However, there is no doubt that some drugs used as anti-depressants – such as fluoxetine - stimulate neurogenesis, particularly the mitotic rate of the progenitor cells.

Chronic, but not acute, treatment of rats with fluoxetine, a typical SSRI (Selective Serotonin Reuptake Inhibitor), stimulates the proliferative rate of the progenitor cells lining the internal layer of the dentate gyrus [12]. This takes around 14 days, which is intriguing since the pharmacological action of this drug begins within hours. These finding suggests that there are downstream actions upon which the proliferativc action of these drugs depends. This latent period recalls the similar one preceding therapeutic effects in patients treated for depression. Santarelli and colleagues reported that suppression of neurogenesis either by radiation or 5HT1A receptor knockout prevented the ameliorating actions of fluoxetine on novelty suppressed feeding [13]. Although this was hailed as support for a link between hippocampal neurogenesis and depression, the behavioural test had more to do with anxiety than depression, and a further study (from the same group) using a different test (forced swim) and a different strain of mouse failed to replicate it [14]. Nevertheless, the necessity for prolonged treatment with drugs such as fluoxetine for both behavioural and cytological actions remains to be explained.

BDNF (Brain-Derived Neurotrophic Factor) is involved in activity dependent neuroplasticity, survival and differentiation in both the periphery and CNS. The BDNF-responsive TrkB (Tropomyosin-associated kinase) receptors upregulate or downregulate many second messengers that lead to the phosphorylation of transcriptional factors such as CREB (c-AMP response element-binding). Chronic antidepressant treatment stimulates pCREB though there have been conflicting reports[15], [16], [17]. Previous studies have also shown that there are increased levels of BDNF protein and mRNA in rat treated with antidepressants [16], [18], [19]. Transgenic mice with reduced BDNF levels or impaired TrkB signalling in the brain have provided evidence that TrkB activation is required for the behavioural actions typically induced by antidepressants [20]. Wnt signalling has been implicated in both embryonic and adult neurogenesis[21], [22], but whether it is involved in the action of fluoxetine on adult neurogenesis is not known.

We have addressed the question of the importance of TrkB receptor activation on antidepressant effect on cell mitosis and on the latent period preceding activation of progenitor cell mitosis. The first experiment determines whether intracerebroventricular (icv) infusion of the Trk antagonist K252a, can block the action of fluoxetine (10 mg/kg/day) on progenitor cell mitosis, and the effect it has on the expression of pCREB and Wnt3a in the granular neurons in the dentate gyrus. The second explores whether rats treated with the same dose of fluoxetine for 7 days show any change in progenitor proliferation, or expression of pCREB or Wnt3a, compared to those treated for 14 days. Then the role of the Trk receptors in the latent period required for fluoxetine to act was investigated by giving K252a for either the first or second 7 days of the 14 day treatment period.

## Materials and Methods

### Animals

All procedures were carried out under Home Office (UK) licence. Male Sprague-Dawley rats 8 weeks old (Harlan, Oxon, UK) were used, weighing 200–250 grams at the start of the experiment. Rats were housed individually in a controlled environment. Ambient temperature was maintained at 21°C and humidity at 55% with *ad libitum* access to food and tap water. Animals were kept in a reversed 12-h light:12-h dark cycle, (lights off at 10.00 h).

### Cannula placement

Animals were anesthetised with isofluorane, oxygen and NO and placed securely into a stereotaxic frame (David Kopf instruments, Tujunga,CA, USA). A cannula (length 5 mm, outside diameter 0.36 mm; (Charles River, Margate, UK) was implanted into the right lateral ventrical. Coordinates were 1 mm posterior and 1.5 mm lateral from bregma, −3.3 mm from the cortex [23].The cannula were fixed in place by dental cement attached to three stainless steel screws inserted into the skull. They were connected to an Alzet osmotic minipumps (model 1002; volume 100 µl, flow rate 0.25 µl/h; (Charles River, Margate, UK) via medical grade vinyl tubing (8 cm length, except in Experiment 2). All pumps were implanted subcutaneously in the posterior upper thorax. The pumps and the tubing had been filled the day before surgery with either K252a (Merck Chemicals, Boulevard Industrial Park, Nottingham) (59.05 ng/day) [24].or saline and incubated at 37°C overnight in a sterile saline solution to prime them before implantation. The position of each cannula was assessed post-mortem by examining its track on sections stained with cresyl violet (Sigma, UK).

### Experimental groups

#### Experiment 1: The effect of K252a on the response to fluoxetine

There were four groups (n = 6 per group). All were implanted with Alzet osmotic mini pumps subcutaneously (model 2ML2; volume 2 ml, flow rate 5 µl/h; Charles River, Margate,UK). In two groups, the pumps delivered fluoxetine (10 mg/kg/day dissolved in saline) for 14 days; in the other two, saline alone. All were also implanted with an icv cannula (right lateral ventricle) connected to a smaller subcutaneous mini-pump (model1002) (see above). One each of the fluoxetine and saline-treated groups received icv K252a 59.05 ng/day[24] for 14 days; the others, saline. The position of each cannula was assessed post-mortem by examining its track on sections stained with cresyl violet (Sigma, UK). All animals were killed on day 15, the brains removed and snap-frozen and placed in −70°C until they were sectioned later. Sections were stained for Ki-67, BDNF and TrkB mRNA by in situ hybridisation, and immunofluorescent for pCREB and Wnt3a proteins.

#### Experiment 2. Blockade of Trk receptors during the initial or later stage of the response to fluoxetine

Two experiments were carried out. In the first, rats (n = 5 per group) were treated either with fluoxetine (10 mg/kg/day) or saline delivered from osmotic minipumps subcutaneously and killed after either 7 or 14 days. Brains were examined for Ki67, pCREB, BDNF and Wnt3a.

In the second, there were four groups (n = 6 per group). All animals received 10 mg/kg/day fluoxetine for 14 days delivered from osmotic minipumps subcutaneously, as above. All were also implanted with a second osmotic minipump, connect to an icv cannula (as above). Through this cannula they received the following treatments: (i) saline 14 days (ii) K252a for 14 days (as above) (iii) K252a for the first 7 days, saline for the second 7 days (iv) saline for the first 7 days, K252a for the second 7 days. The osmotic minipumps were attached to the tube 11.3 cm long and a volume of 3.74 ml/cm (ID 0.69 mm). Since flow is 6 ml/day, the tube will empty by day 7. This was checked in vitro at 37°C. In group (ii) both tubing and pump were filled with K252a; in group (iii) the tubing contained K252a, and the pump saline only; this was reversed in group (iv). All animals were killed on day 15 and the brains were snap frozen and placed in −70°C until they were sectioned (as above). Sections were stained for Ki-67.

### Brain sections

Brains were sectioned in the coronal plane at 20 µm. The brain from each rat was sampled from −2.80 to −4.52 behind bregma. Sections were cut at −20°C using a Bright cryostat, and every sixth section was mounted on a polylysine-coated microscopic slide, six to a slide. The slides were left in a fume hood to dry overnight, then stored at −70°C. For each rat twelve sections through the dorsal hippocampus were analyzed 120 µm apart (ie one in six sections) for Ki-67 staining.

### Immunohistochemistry

#### Ki67

Sections were incubated in 0.01 M citric acid for 40 min at 98°C. They were cooled and washed twice ×5 min in KPBS. Endogenous peroxidase activity was quenched with 3% H2O2 solution for 10 min followed two 5 min washes with KPBS. They were incubated with primary antibody (1∶100 mouse monoclonal IgG anti-human Ki-67; Novocastra, Newcastle Upon Tyne, UK) and 1% horse serum in a humidified chamber at room temperature overnight. The next day, after twice ×5 min washes with KPBS, they were incubated with secondary antibody (1∶200 biotinylated mouse IgG; Vector laboratories Ltd, Peterborough UK) for 1 hour at room temperature. After twice ×5 min washes with KPBS, they were incubated with Avidin-Biotin-Peroxidase reagent (Vector laboratories Ltd, Peterborough UK) for a further hour, followed by twice ×5 min washes with KPBS. The staining was visualized using DAB tablets (3,3-diaminobenzidin) (Sigma, Dorset, UK) for 5 min. Slides were then counterstained with 10% crystal violet solution followed by dehydration through ethanol and Histoclear. They were cover-slipped with DPX for light microscopy at ×40 magnification.

#### pCREB and Wnt3a

Three sections from each animal (1 in 12; between bregma −3.14 mm and −3.30 mm) were incubated in rabbit anti pCREB (Cell Signaling, Hitchen, UK; 1∶25 in 0.5% Triton with 1% goat serum) overnight in a humidified chamber at room temperature. After two washes with KPBS, the sections were incubated in Alexa Fluor 568 (Invitrogen, Paisley UK) goat anti rabbit (1∶200 in 0.5% Triton) for one hour, washed twice with KPBS, and cover slipped.

For single staining with Wnt3a, sections were incubated in rabbit anti Wnt3a (Abcam, Science Park, Cambridge UK; 5 ug/ml in 0.5% Triton with 1% goat serum) overnight in a humidified chamber at room temperature. The following day, after two washes with KPBS, they were incubated in Alexa Fluor 568 goat anti rabbit (1∶200 in 0.5% Triton) for one hour, and washed twice with KPBS and covered slipped.

### BDNF mRNA in situ hydridisation

Sections were allowed to air dry at room temperature and were then fixed with 4% paraformaldehyde (Sigma, Dorset, UK) for 5 min, washed in PBS and then dehydrated in 70% ethanol and 95% ethanol for 5 min before final storage in fresh 95% ethanol. In situ hybridization was carried out under RNAase-free conditions. The synthetic antisense oligonucleotide probe was confirmed by BLAST searches. A 48 base and a 45 base oligonucleotide complementary to exonic mRNA encoding BDNF mRNA [25]. 5′ agt tcc agt gcc ttt tgt cat gcc cct gca gct tcc ttc gtg taa ccc ′3 was used. All probes were end-labelled with ^35^S-ATP as follows: 2 µl of purified oligonucleotide (5 ng/µl) was added to 1.25 µl Buffer and 1.25 µl cobalt chloride (New England, Biosystem, UK). DEPC (Diethyl pyrocarbonate Sigma, Dorset, UK)-treated water (6.5 µl) was added, followed by 1 µl terminal 35S deoxyadenosine 5′ (α-thio) triphosphate (10 mCi/ml) (Amersham, Buckinghamshire, UK) and 0.5 µl (15–20 U) terminal deoxynucleotide transferase enzyme (New England, Biosystem UK). Probes were incubated at 37°C for 1 h before 40 µl of DEPC was added to terminate the reaction. Purification of labelled probe from unincorporated nucleotides was accomplished by centrifugation through a G-50 Sephadex micro-column (Amersham, UK). Probes were evaluated for incorporation of radiolabel by scintillation counting. All hybridizations were carried out at 2500–5000 cpm/µl in hybridization buffer (50% deionized formamide, 4× SSC, 5× Denhardt's,100 µg/ml polyadenylic (potassium salt) acid, 200 µg/ml salmon sperm DNA, 120 µg/ml heparin (BDH, Leicestershire, UK), 25 mM sodium phosphate pH 7.0,1 mM sodium pyrophosphate, 10% (w/v) dextran sulphate in DEPC–treated water (all Sigma Dorset, UK). Sections were covered with parafilm and hybridized overnight at 44°C in a humid atmosphere. Excess unbound probe was removed using the following washes: 1× SSC (saline sodium citrate, Sigma, UK) at room temperature, 2×30 min at 55°C with 1× SSC and then rinsed at room temperature for 2 min, each in 1× SSC, 0.1× SSC, 18 Ω water, 50%, 70%, and 95% ethanol (BDH. Leicestershire, UK).

Sections were exposed to autoradiographic X-ray film (Amersham, Buckinghamshire, UK) for 14 days. Sense probes were run as negative controls.

### Quantification

#### Proliferating cells (Ki67)

All slides were randomised and coded prior to quantitative analysis.

Labelled cells were counted using a 40X objective; only cells on the internal border of the subgranular zone of the dentate gyrus were included. The data shown are the mean number of Ki67-labelled cells per section from 12 sections per animal.

#### BDNF mRNA expression

Sections and C^14^ labeled standards of known radioactivity (Amersham, Buckinghamshire, UK) were placed in X-ray cassettes and exposed to autoradiographic film. The optical density (OD) of the autoradiographic images was measured using a computerized PC-based image analysis system (NIH Image). ODs from the dentate gyrus from three consecutive sections per rat were obtained and averaged. The mean value for each rat was entered into the equation derived from the C^14^ standards and the final value were used to calculate group means. Sections from all groups were processed at the same time to avoid intrinsic variations between different in situ hybridizations.

### Statistical analysis

Between-group one/two-way analysis of variance (ANOVA) and Bonferroni's post hoc test were used when applicable. Log transformation was used to ensure homogeneity of variance before ANOVA when appropriate. Results were considered statistically significant if p<0.05.

## Results

### Experiment 1: Effect of K252a on the response of progenitor cells to fluoxetine

In this experiment, we tested whether blockade of the Trk receptor by icv K252a would prevent the stimulating action of fluoxetine on mitosis in the neurogenic area of the dentate gyrus.

#### Ki-67

**Figure 1:**
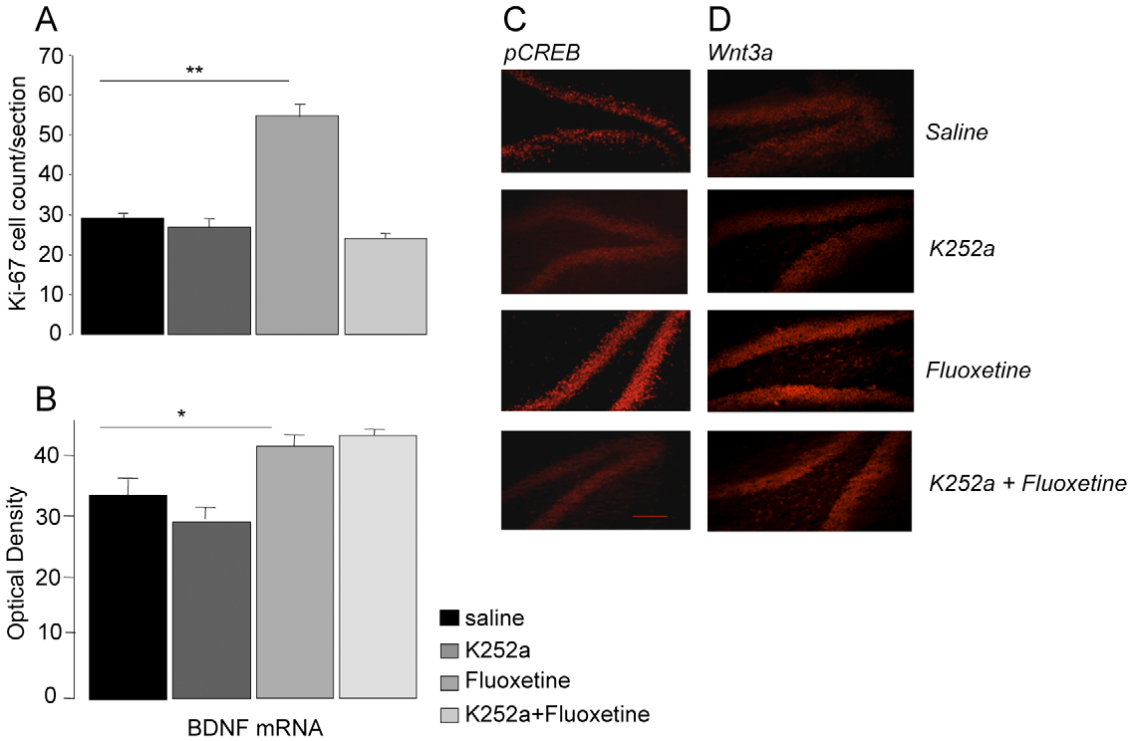
The effect of K252a icv on the mitosis rates (Ki67) of progenitor cells in the dentate gyrus. A: Mean number per section after 14 days treatment with either fluoxetine (10 mg/kg/day) sc, K252a icv, or both compared to controls (saline sc and icv). Values are means ± SEM. C,D: Photomicrographs of the expression of pCREB and Wnt3a after the four treatments. Bar represents a distance of 100 µm. B; BDNF mRNA in the dentate gyrus following the same treatment. Values are mean ±SEM. *P<0.05, **p<0.001 compared to control.

Ki67 positive cells were found in the SGZ of all animals including controls, confirming the presence of a basal level of progenitor cell mitosis in this region. There was a significant interaction between fluoxetine and K252a treatments (Two-way ANOVA, F (1,20) = 36.7, p<0.001). Icv K252a alone had no effect on the number of proliferating cells (Bonferroni, p>0.05). 10 mg/kg/day fluoxetine increased the number of proliferating cells by 88% (p<0.001) (Figure 1A). This effect was completely blocked by K252a (p>0.05). The number of Ki67-labelled cells in this group was not significantly different from the controls group (p>0.05), but was significantly less than after fluoxetine treatment alone (p<0.005) (Figure 1A).

#### BDNF mRNA

K252a alone had no effect (F = 0.28; p:ns). Fluoxetine increased levels, as expected (F = 34.6. p<0.0001), but this was not prevented by simultaneous infusion of K252a; there was thus no significant interaction between K252a and fluoxetine (F = 2.4, p:ns) (Figure 1B).

#### pCREB

Saline-treated animals showed little expression of pCREB protein in the dentate gyrus. This was markedly stimulated by fluoxetine treatment. K252a-treated animals showed a similar picture to the saline-treated group (Figure 1C). Adding K252a to fluoxetine resulted in reduced pCREB expression which now resembled animals treated with saline.

#### Wnt3a

Saline-treated animals showed little expression of Wnt3a protein in the dentate gyrus (Figure 1D). This was markedly stimulated by fluoxetine treatment. K252a-treated animals showed a similar picture to the saline-treated group. However, adding K252a to fluoxetine did not prevent the latter's stimulating effect on Wnt3a expression (Figure 1D).

In summary: this experiment showed that icv K252a prevented the stimulating effect of fluoxetine on progenitor mitosis in the dentate gyrus, but did not prevent increased BDNF mRNA. There was a selective action of K252a on inhibiting the effect of fluoxetine on pCREB but not Wnt3a.

### Experiment 2: Effect of treatment with K252a for either the first or second 7 days of a 14 day fluoxetine treatment

Since fluoxetine treatment is required for at least 14 days to increase progenitor cell mitosis rates, we asked whether activation of Trk receptors was required for the whole of this period.

#### Ki-67

**Figure 2:**
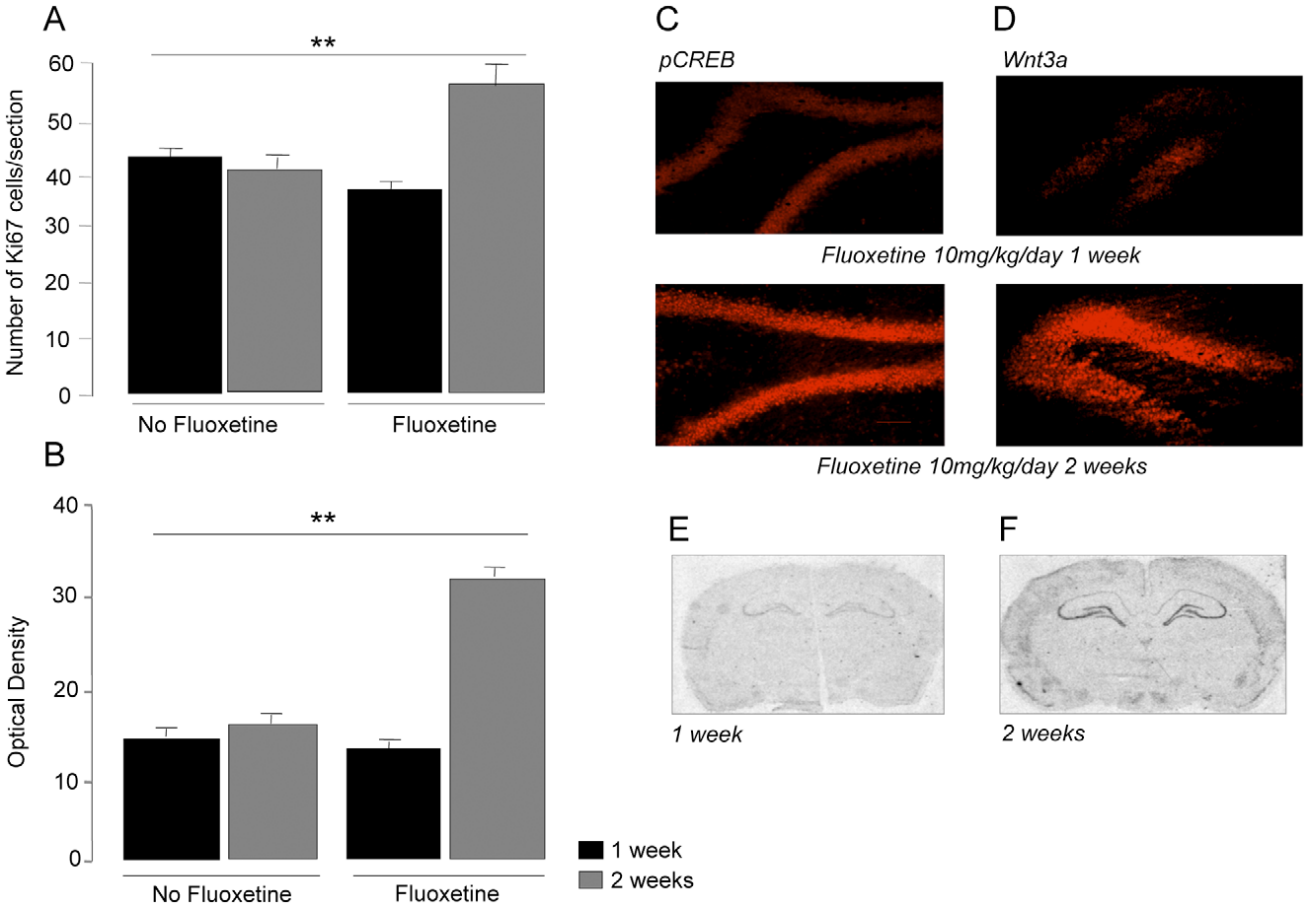
The effect of either 7 or 14 days treatment with fluoxetine (10 mg/kg/day). A: Ki67 cell counts, B: BDNF mRNA expression, C: pCREB D: Wnt3a. E,F: *in situ hybridization* images of BDNF mRNA. Values as in Figure 1. Bar represents 100 µm. Values are mean ±SEM.**p<0.001 compared to control. Representative sections are shown from fluoxetine-treated animals only, since there is virtually no expression in controls.

The number of Ki-67 cells increased significantly as expected after 14 days of fluoxetine treatment (Figure 2A). However, at 7 days there was no difference between fluoxetine and control groups (two-way ANOVA: fluoxetine × time F = 14.0, p = 0.002).

#### BDNF

BDNF mRNA expression showed a similar pattern. Expression increased after 14 days in the fluoxetine-treated group, but not 7 days, (F = 150, p<0.001) (Figure 2B). Pairwise comparisons showed that BDNF levels at 7 days were no different between the fluoxetine-treated and control groups (Bonferroni, p>0.05) but that fluoxetine treatments at 7 and 14 days were significantly different from each other (p<0.001) (Figure 2E, F).

#### pCREB and Wnt3a

There was also no observable change in either pCREB or Wnt3a at 7 days, but by 14 days the expression of both was clearly increased (Figure 2C, D).

#### Ki-67

**Figure 3:**
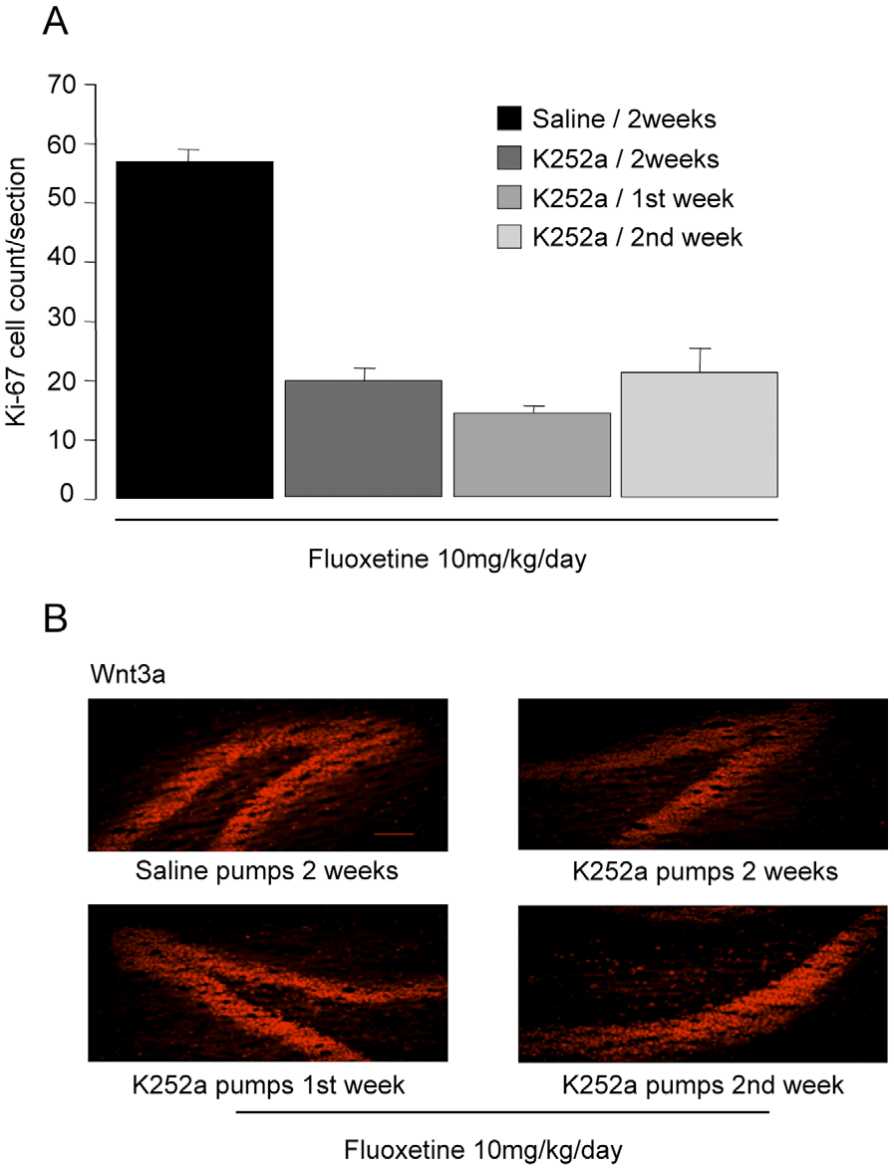
Effect of treatment with K252a icv for either the first or second 7 days of a 14 day fluoxetine (10 mg/kg/day) treatment. A: Ki67-labelled cell counts; B: Wnt3a expression in the dentate gyrus. Values as in Figure 1. Bar represents 100 µm.

In the second part of this experiment, as expected from Experiment 1, icv K252a for 14 days reduced Ki67 positive cells expression in 14-day fluoxetine -treated rats compared to those receiving fluoxetine and icv saline (One-way ANOVA: F (3,15)  = 63.8, p<0.001. Pairwise comparison: (Bonferroni) p<0.001). However, K252a infusions for either the first or second 7 days also reduced the effect of 14 days fluoxetine treatment, and there was no difference between the two 7-day treatment periods (Bonferroni, p>0.05) There was also no difference between either 7-day and 14 day K252a treatment (Bonferroni, p>0.05) (Figure 3A). As in Experiment 1, pCREB expression was inhibited by 14 days K252a treatment (not shown in Figure 3, since there was effectively no signal in the K252a treated animals) but Wnt3a expression was not altered, even though Ki67 expression was reduced (Figure 3B).

#### In summary

The first part of this experiment showed that 7 days treatment with fluoxetine had no effect on Ki67 labelled cells, or on the expression of either pCREB or Wnt3a. However, all three were accentuated after 14 days treatment. The second part showed that blocking Trk receptors with icv K252a for either the first of second 7 day period of a 14 day fluoxetine infusion prevented the expected increase in progenitor cell mitosis. As in experiment 1, pCREB was reduced in fluoxetine and K252a-treated animals, but increased expression of Wnt3a still occurred in fluoxetine-treated animals given K252a.

Table 1 summarises the results of these two experiments on the expression of Ki67, pCREB and Wnt3a in the dentate gyrus.

**Table 1:** Summary of the effects of fluoxetine (flx), and K252a on the expression of Ki67, pCREB and Wnt3a in the dentate gyrus of the adult male rat.

Treatment	BDNF mRNA	TrkB mRNA	Ki67	pCREB	Wnt3a
Flx (10 mg/day 14 days)	1	0	1	1	1
K252a 14 days	0	0	0	0	0
Flx (10 mg/day 14 days) + K252a	1	0	0	0	1
Flx (10 mg/day 14 days)	1	0	1	1	1
Flx (10 mg/day 7 days)	0	0	0	0	0
Flx (10 mg/day) + K252a (days1–14)			0	0	0
Flx (10 mg/day) + K252 (days1–7)			0	0	0
Flx (10 mg/kg/day)+ K252a (days7–14)			0	0	0

0 =  no increase compared to control; 1 =  increased compared to controls.

## Discussion

These experiments further define the role of BDNF in the regulation of the mitosis rates of progenitor cells in the dentate gyrus by serotonin-acting drugs such as fluoxetine. Progenitor mitosis rates are the essential first step in the overall control of neurogenesis in the adult hippocampus, since they determine its early stages. Other factors then regulate the differentiation, survival and connectivity of newly-formed neurons [26]. Labelling cells with Ki67 is now well established as a reliable indicator of progenitor cell mitosis in the dentate gyrus, and is highly correlated with BrdU uptake [27]. The first experiment reported here established that blockade of Trk receptors by the drug K252a was able to prevent entirely the stimulating action of fluoxetine on mitosis of the progenitor cells. The K252 family of alkaloid toxins are protein kinase inhibitors, and K252a has a long history of being used to antagonise Trk receptors, with particular affinity for TrkB [28], [29], [30]. Icv K252a has already been shown to prevent some actions of BDNF on serotonin release after SSRI administration [31], but there has been no report on its ability to inhibit SSRI-induced progenitor cell mitosis. Although we cannot be entirely certain that our results reflect specific blockade of TrkB, the accumulative evidence points strongly in this direction.

K252a did not prevent the fluoxetine-induced increase in BDNF mRNA expression, suggesting that this is upstream of the action of TrkB receptors, as might be expected. However, the results on pCREB were clear-cut: fluoxetine increased its expression and thiswas prevented by K252a. Since increased pCREB may be an essential step in the up-regulation of progenitor mitosis [32], it is interesting that this occurred not just in the cells adjacent to the neurogenetic niche in the inner layer, but throughout the dentate gyrus. This suggests that the function of CREB in the dentate gyrus may be more complex than was originally thought, and that cells other than those in the neighbourhood niche may also contribute to the control of neurogenesis [7]. In contrast, the expression of Wnt3a, also known to be closely involved in neurogenesis [3], [22], [33], [34] was, as expected, increased by fluoxetine, but this was not prevented by K252a. This experiment therefore shows that functional activity of Trk, and hence – it may be presumed – BDNF is essential for the stimulating action of fluoxetine on progenitor cell mitosis. This agrees with results on BDNF-knockout mice, in which SSRIs and similar drugs have reduced actions [35], [36]. However, these studies were limited to heterozygous knock-outs only, since homozygous ones do not survive. It is interesting that K252a by itself did not decrease progenitor mitosis below baseline: this may reflect the fact that its blockade of TrkB may have been incomplete, or that BDNF is required only for above-basal proliferation rates. K252a also blocked the fluoxetine-induced expression of pCREB, supporting the role for this compound in the control of neurogenesis. However, it did not prevent increased Wnt3a expression, suggesting that the latter is not a sufficient event for increased progenitor cell mitosis.

The second experiment began by confirming, under our conditions, that a 7-day treatment with fluoxetine had no discernible effect on progenitor mitosis, or pCREB and Wnt3a expression, whereas after 14 days all three were stimulated. We use osmotic minipumps to deliver fluoxetine, since we (and some others) find that parenteral injections are not effective [37], [38]. The striking result, however, was that blocking TrkB for either the first or the second 7 days of a 14 day treatment with fluoxetine prevented increased progenitor mitosis. It is during the second period that BDNF mRNA expression is increased, so it might seem logical that blockade only during this time would prevent the BDNF-dependent action of fluoxetine. Had this occurred it would have implied that the latent period preceding the onset of fluoxetine's action was due to some event upstream of the action of BDNF. But this was not the case. It seems clear that BDNF is required throughout the latent period, though future experiments might explore this more precisely, using more frequent sampling points. Despite the fact that BDNF mRNA was increased only after 14 days of fluoxetine treatment, blocking its action at a time when there was no discernible change in BDNF mRNA prevented not only the expected increase in progenitor mitosis, but also that in pCREB, presumably a more proximal regulator of neurogenesis. The conclusion must be that BDNF may be active throughout the latent period. However, we have shown previously that icv infusions of BDNF are effective after 7 days [39], so the latent period is not due to a delayed response to BDNF. Our experiments do not yet allow us to offer a precise explanation of these results, but there may be two processes. The first, dependent on ‘basal’ BDNF, may represent one that, in some way, allows increased BDNF to occur at some time after 7 days from initiation. The second part of this process may depend on heightened BDNF production, and it is this that results in increased pCREB expression, and thus enhanced progenitor mitosis. Some part of this whole process is highly sensitive both to the absolute levels of corticoids as well as their circadian pattern [26], [39], [40], [41], [42].

Our results suggest that the expression of pCREB is downstream of the action of BDNF, since it was prevented by K252a. Since CREB has a range of intracellular actions, these may not all link with Wnt3a. Wnt3a has been implicated in the ability of human neural progenitors to form neurospheres in culture [33] and in the process of adult neurogenesis, though its exact role is still unclear [43], [44]. Our results suggest that factors additional to Wnt3a (maybe other members of the Wnt family) may be needed to increase progenitor cell mitosis rates. Table 1 shows that, whilst we find a consistent association between pCREB and increased mitosis, this was not true for Wnt3a. However, increased Wnt3a may be an essential ingredient of the stimulatory process, since we never observed one without the other, and inhibition of Wnt signalling reduces adult neurogenesis in the dentate gyrus [3]. Our results further suggest that the stimulating action of fluoxetine on Wnt3a may not be dependent on BDNF (or Trk receptors: we have not investigated the lower-affinity p75 receptor), so there may be other pathways linking the action of this drug to neurogenesis in the dentate gyrus. The ability of fluoxetine to increase Wnt3a is consistent with this drug's reported induction of neural plasticity [45], [46], [47]. This may well contribute to its efficacy as an anti-depressant.

The experiments reported here emphasise the central role of BDNF in the regulation of mitosis of the progenitor cells in the dentate gyrus of the adult rat, and its contribution in the latent period preceding the effect of fluoxetine on this process. The role of BDNF in the expression of pCREB and Wnt3a may be distinct. Our results further suggest that the control of the neurogenetic niche may be widespread throughout the dentate gyrus, though the identity of the cells that produce BDNF is still obscure. They do not add directly to the evidence linking adult neurogenesis with depression, but strengthen understanding of the role of BDNF and its receptor in the action of anti-depressants such as fluoxetine.
